# Relationship among the COM Motion, the Lower Extremity and the Trunk during the Squat

**DOI:** 10.5114/jhk/183066

**Published:** 2024-05-17

**Authors:** Satoshi Kasahara, Tomoya Ishida, Jiang Linjing, Ami Chiba, Mina Samukawa, Harukazu Tohyama

**Affiliations:** 1 Department of Rehabilitation Sciences, Faculty of Health Sciences, Hokkaido University, Sapporo, Japan.; 2 Department of Rehabilitation, Hirosaki University Hospital, Hirosaki, Japan.

**Keywords:** squatting, kinesiology, center of mass, knee, postural control

## Abstract

Squatting is a common motion in activities of daily living and is frequently used in training programs. Squatting requires a shift of the body in both vertical and anterior-posterior directions. Postural control during squatting is considered a mixed strategy; however, details and roles of the trunk and lower limb joints are unclear. The purpose of this study was to investigate the relationship among the kinematics of the lower limb, the trunk and the center of mass (COM) descent during squatting. Twenty-six healthy young adults performed repeated parallel squats. Lower limb joint and trunk angles and the COM were analyzed using a 3D motion analysis system. We evaluated the relationship between the kinematics and the squat depth by performing correlation analysis and multiple linear regression analysis. The ankle was the first to reach its maximum angle, and the remaining joints reached their maximum angles at the maximum squat depth. The knee joint motion and the squat depth were significantly correlated and there was a correlation between the hip and the ankle joint motion and the anteroposterior displacement of the COM during squatting. Multivariate linear regression analysis indicated that squat depth was predicted by both the knee and ankle motion and that anteroposterior displacement of the COM was predicted by the hip, ankle, and knee joint motion. The knees contributed to the vertical COM motion during squatting, while the hips contributed to the COM motion in the anteroposterior direction. On the other hand, the ankles contributed to COM motions in both the vertical and anteroposterior directions during squatting.

## Introduction

Squatting is a basic skill, and its variations are used to accomplish various tasks associated with activities of daily living (e.g., descending stairs, sitting down, and using the toilet) (Flanagan et al., 2003; Kuo et al., 2011). In addition, squats are widely used as a typical exercise to improve and strengthen lower limb function in musculoskeletal rehabilitation and sports (Escamilla et al., 2001). However, because the postural strategy during squats is much more complex and difficult than that during upright standing, numerous studies are underway to investigate the kinematics and kinetics of squats.

A unique feature of squats compared to other postural strategies is that they lower the center of mass (COM), and the kinematics and kinetics in both the anteroposterior (AP) and vertical directions need to be better understood (Flanagan et al., 2003; Kasahara et al., 2015; Nashner et al., 1985; Oude Nijhuis et al., 2007). Most studies have primarily focused on joint motions of the lower limbs in the sagittal plane and the center of foot pressure (COP) motion in the AP direction (Dionisio et al., 2008; Escamilla et al., 2001; Hase et al., 2004; Kim et al., 2014). In line with the postural control theory, that is, the ankle or hip postural strategy (Kasahara et al., 2015; Nashner et al., 1985), the ankle is considered to contribute to the COP shift in the AP direction during squatting (Dionisio et al., 2008). Moreover, Hase et al. (2004) revealed that the initial COP motion in the AP direction during squatting depends on postural muscles released around the ankle joint. In contrast, information regarding the contribution of the trunk or the hip joint to COP or COM motions during squatting is insufficient (Kim et al., 2014; Schoenfeld, 2010).

Although squatting is characterized by downward movements, as mentioned above, measures of quantification of squat performance (i.e., squat depth) have been inconsistent among studies (Schoenfeld, 2010). Generally, squats are indirectly measured by the degree of flexion at the knee (Schoenfeld, 2010) or a marker placed on a certain body part (Bagwell et al., 2016; Kim et al., 2015; Zawadka et al., 2020). As these variables depend on other joint motions and segment positions, such joint motions and the coordination among joint motions of the lower limbs must be determined. Therefore, kinematic or kinetic variables (e.g., the COM position) other than the joint motion are necessary for reliable assessment of squatting ability (Bagwell et al., 2016; Schoenfeld, 2010; Stevens et al., 2018; Zawadka et al., 2020). In addition, several reports on the relationship between squat depth and lower limb joint angles have been published; however, participants in those studies were instructed to squat deeply with maximum effort, and the criteria for and values of the squat depth differed among the participants (Kim et al., 2015; Zawadka et al., 2020).

Squat, which is a mixed or a suspensory strategy, is reportedly related to falls (Sugama et al., 2020) and balance ability (Kasahara et al., 2015). However, the intrinsic role of each lower limb joint during squats remains unclear. The aim of this study was to investigate the relationship between the kinematics and COM displacement (i.e., both vertical and AP postural control) in the sagittal plane during squats. For this study, we defined the squat depth using the vertical height of the COM and a three-dimensional body segment model. We hypothesized that, although the existing literature (Kasahara et al., 2015; Kim et al., 2015; Zawadka et al., 2020) indicates that all joints contribute during the squat, the knee would especially contribute to vertical postural control, and the ankle and hip joints would be recruited for AP postural control.

## Methods

### Participants

Twenty-six healthy college students (13 men and 13 women, mean ± standard deviation age 22.6 ± 1.2 years, body height 1.67 ± 0.08 m, body mass 58.3 ± 9.5 kg) participated in this study. The adequacy of the sample size and the significance level were confirmed using G*Power (Faul et al., 2007), with an effect size of 0.6, alpha of 0.05, and power of 0.8, according to Cohen’s criteria (Cohen, 1988). All participants lived independently in their community without problems regarding activities of daily living and had no disorders, injuries, or any neurological, vestibular, orthopedic, or cognitive conditions that could interfere with their balance. This study was conducted in accordance with the principles embodied in the Declaration of Helsinki. All participants provided written informed consent and the study procedures were approved by the Institutional Review Board of the Faculty of Health Sciences, Hokkaido University (approval number: 19-72; approval date: 12 December 2019).

### Experimental Approach

**Figure 1:**
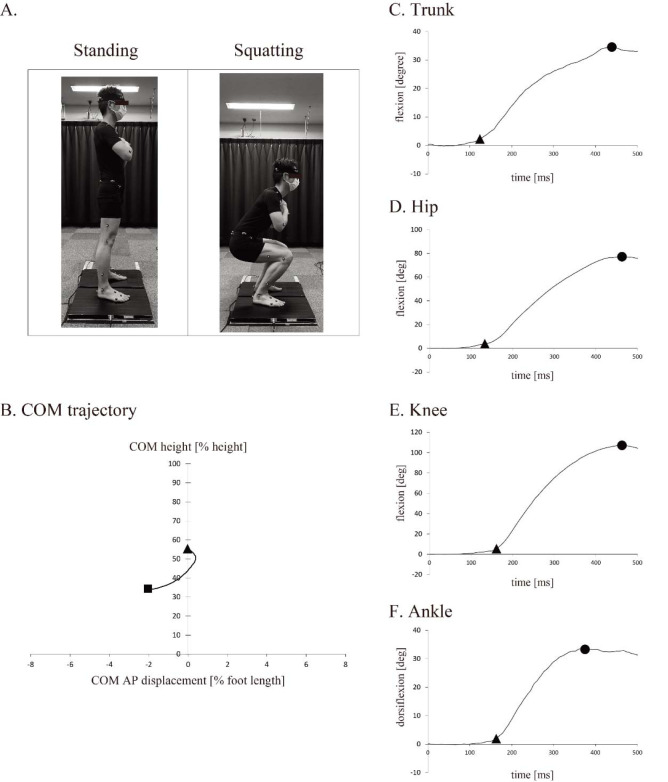
A. Experimental setup of the squatting, B. Typical COM trajectory in the sagittal plane from one participant (participant no.14). Triangle and square markers indicate the start and the deepest position, respectively. The positive and negative signs in the X axis indicate the anterior and the posterior direction, respectively. Panels C, D, E, and F present the typical change of each joint angle in the sagittal plane (participant no.14). The positive value in panels C, D, and E is flexion and the positive value in panel F is dorsiflexion. Triangle and circle markers indicate the onset and the maximum joint angle, respectively.

Participants were asked to assume a relaxed, upright posture with their feet shoulder-width apart and arms crossed over their chest, and then, without their heels leaving the ground, to squat until their thighs were parallel to the floor, for 2 s (Figure 1A) (Ishida et al., 2022b; Webster et al., 2015). Participants, facing anteriorly, returned to the original upright posture after the squat and repeated the squat five times. No instruction or feedback information was provided to participants, except for those mentioned above.

A three-dimensional motion analysis system (Cortex 5.0.1.; Motion Analysis Corp., Santa Rosa, CA, USA) with seven near-infrared cameras (Hawk cameras; Motion Analysis Corp.) was used to obtain marker trajectory data during the squat. The sampling rate was set to 200 Hz. A total of 37 retroreflective markers were placed on the anterior and posterior sides of the head, shoulders, elbows, wrists, sternum, and the iliac crest; as well as the anterior and posterior sides of the iliac spine (ASIS and PSIS), the lateral side of the thighs, the medial and lateral sides of the femoral epicondyles, the lateral side of the shanks, the medial and lateral sides of the malleoli, and the second metatarsal heads and bases, fifth metatarsal heads, and heels. All markers were recorded data during the squats and stored them on a PC for further analysis.

### Data Processing and Analysis

Three-dimensional trunk, hip, knee, and ankle kinematics were estimated in the sagittal plane using a rigid-body skeletal model with a joint coordinate system (Visual 3D version 6; C-Motion Inc., Germantown, MD, USA). The trunk flexion angle was defined as the orientation of the thoracic segment relative to the laboratory coordinate system. The lower limb joint angle was calculated for each joint coordinate system using the Cardan X-Y-Z sequence. The marker trajectory data were low-pass filtered using a zero-lag, fourth-order Butterworth filter with a cut-off frequency of 12 Hz (Ishida et al., 2022a). The trajectory gap of the ASIS markers was filled based on the iliac crest and PSIS markers (McClelland et al., 2010). The COM position of the whole body was calculated from the COM position of each segment, which was estimated based on a previous report (de Lev et al., 1996). COM displacements in the vertical (descent) and AP (AP displacement) directions were normalized to the participants’ height and foot length, respectively, and the values were presented as percentages. Positive values of calculated joint angles indicate flexion and dorsiflexion, and negative values indicate extension and plantar flexion. Additionally, the anterior and posterior directions are indicated as positive and negative, respectively.

All data analyses were performed using a customized program in MATLAB (MathWorks Inc., Natick, MA, USA) (Kasahara et al., 2015). Each of the five consecutive squats was divided into separate components of motion. The onsets of the COM descent and motion of each joint were defined as the time when the measured value changed by 5% of the maximum value from the baseline. Furthermore, joint motion onsets were recalculated at time zero as a reference for the COM onset (Figure 1B). Thus, a negative value indicates that joint motion started earlier than the COM descent. Additionally, the time required to reach the maximal angle of each joint (peak time) was measured, and the time difference to the time of the maximal COM descent was recalculated. The maximum squat depth was calculated as the amplitude difference between the highest and deepest positions of the COM, and the AP displacement was calculated as the position change from the start to the final position of the COM. The range of motion (ROM) of each joint was calculated as the difference between the onset angle and the maximal degree (Figure 1C–F).

### Statistical Analysis

Statistical analyses were performed using JMP Pro version 15.2.0 (SAS Institute Inc., Cary, NC, USA). All data are shown as means ± standard deviations. The normality of the data distribution was examined using the Shapiro-Wilk test. For normally distributed data, a repeated-measures one-way analysis of variance (ANOVA) was used to assess the differences among joints, followed by a Bonferroni post hoc test. For non-normally distributed data, if the Freidman test revealed a significant difference, a Wilcoxon signed-rank post hoc test was performed. Pearson’s correlation analysis (R) was performed to assess the relationship between the ROM of each joint and squat depth, and between the joints. After confirming the absence of multicollinearity by a variance inflation factor < 5 (Akinwande et al., 2015), multivariate regression analysis was performed using backward stepwise selection based on the minimum Akaike information criterion to identify whether any of the joints could explain the squat depth (a dependent variable). The effect size was calculated as partial eta-squared values (η2) for repeated-measures one-way ANOVA, and as r values for the Wilcoxon signed-rank test (Cohen, 1988). All significance levels were set at *p* < 0.05.

## Results

### Maximal Squat Depth and ROM of Each Joint

A typical COM trajectory is shown in Figure 1B. Most of the participants shifted their COM forward and backward. The maximal squat depth was 23.1% ± 2.5% of the participant’s height during the squat in this experiment, and the AP displacement of the COM was −5.0% ± 9.6% of the participant’s foot length. ROMs of the trunk, hip, knee, and ankle joint were 34.7° ± 11.5°, 82.7° ± 6.1°, 107.2° ± 11.1°, and 32.8° ± 5.6°, respectively. The ratios of each joint angle to squat depth were as follows: trunk, 1.5° ± 0.5°/%; hip, 1.5° ± 0.5°/%; knee, 4.6° ± 0.3°/%; and ankle, 0.7° ± 0.1°/%.

The onset time did not significantly differ among the joints (F(1.60, 39.86) = 1.087, *p* = 0.334, η^2^ = 0.042), and the onset time of all the joints was earlier than the onset of the COM descent (Table 1). Significant differences were observed in the time to reach the maximal angle among the joints (F(3, 75) = 7.993, *p* = 0.001, η^2^ = 0.242). The post hoc tests revealed that the peak time for the ankle was significantly earlier than those for the knee (*p* = 0.003) and the hip (*p* = 0.002) (Table 1). In addition, the peak times did not significantly differ between the trunk and lower limb joints, or between the hip and knee joints.

**Table 1:** Comparisons of the onset and peak time between joints.

Measurements	Trunk	Hip	Knee	Ankle
Onset time [ms]	−54.5 ± 23.7	−59.2 ± 17.8	−51.8 ± 19.5	−53.2 ± 29.5
Peak time [ms]	−11.5 ± 23.6	−1.1 ± 8.6	0.4 ± 3.0	−21.0 ± 26.6^a,b^

Values are presented as mean ± standard deviation. a is a significant difference from that of the hip joint (p < 0.05). b is a significant difference from that of the knee joint (p < 0.05)

### Correlations

**Figure 2:**
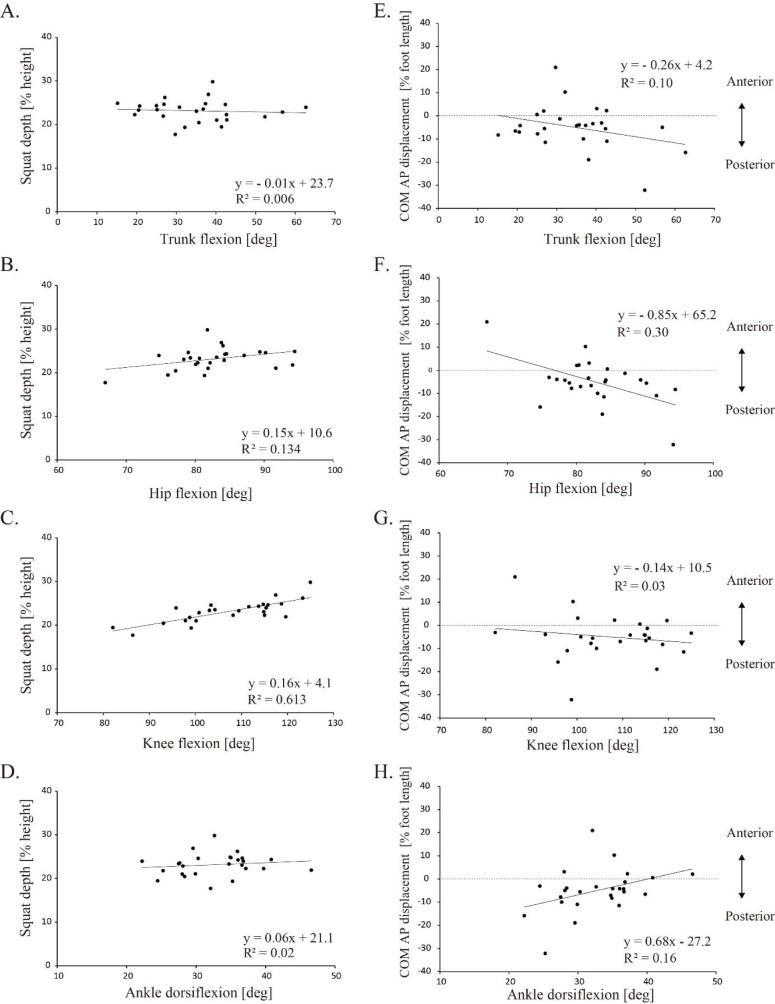
The scatter plot and the regression line for squat depth and the center of mass (COM) versus the joint angle.

Correlation analysis showed that the knee ROM had a significant positive correlation with squat depth (knee: R = 0.783, *p* < 0.001). However, no significant correlation was observed between squat depth and trunk, hip or ankle ROM (trunk: R = −0.071, *p* = 0.729; hip: R = 0.368, *p* = 0.064; ankle: R = 0.134, *p* = 0.514) (Figure 2A–D). In contrast, AP displacement was significantly positively correlated only with ankle ROM (hip: R = −0.538, *p* = 0.005; ankle: R = 0.398, *p* = 0.044), and not significantly correlated with trunk or knee ROM (trunk: R = −0.316, *p* = 0.115; knee: R = −0.167, *p* = 0.413) (Figures 2E–H). Table 2 shows the results of the multivariate linear regression analysis for each joint according to squat depth. Both the knee ROM (explained variable: 59.7%) and ankle ROM (27.1%) were predictors of squat depth (adjusted R^2^ = 0.868, *p* < 0.001), and the hip ROM (26.0%), ankle ROM (16.7%), and knee ROM (12.7%) were significant predictors of AP displacement of the COM (adjusted R^2^ = 0.607, *p* = 0.012) (Table 2).

**Table 2:** Stepwise multivariate linear regression models predicting the squat depth and COM AP displacement for squatting.

	*B* (95% CI)	Standardized *β*	*p* value	VIF
Squat depth				
Knee ROM	0.282 (0.236 to 0.328)	1.240	< 0.001	1.787
Ankle ROM	−0.310 (−0.401 to −0.220)	−0.689	< 0.001	1.787
AP displacement				
Hip ROM	−0.601 (−1.088 to −0.113)	−0.381	0.018	1.247
Ankle ROM	1.335 (0.677 to 1.993)	0.781	< 0.001	1.928
Knee ROM	−0.471 (−0.827 to −0.114)	−0.546	0.012	2.220

B unstandardized regression coefficient, CI confidence interval, VIF the variance inflation factor

### Coordination among Related Joints during the Squat

**Figure 3:**
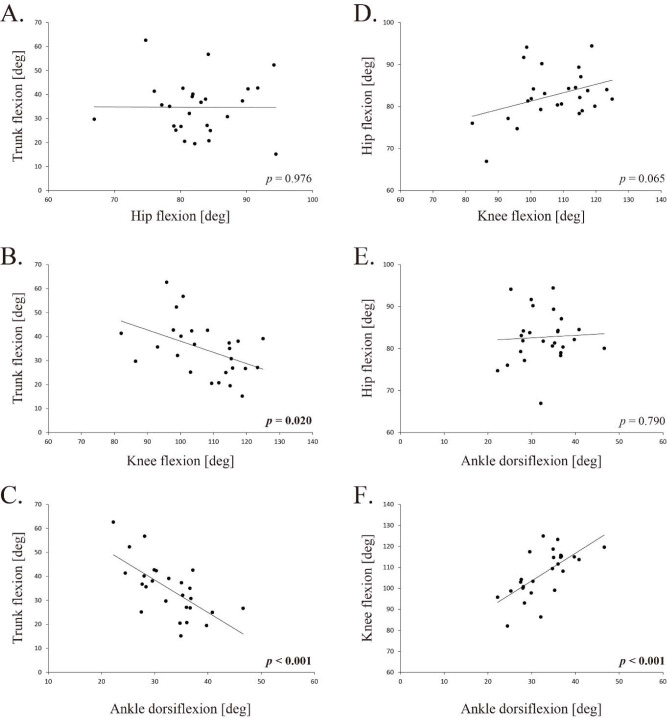
The Pearson correlation among ROMs.

Following the Pearson’s correlation analysis, significant negative correlations were observed between the trunk and the knee ROM (R = −0.452, *p* = 0.020, Figure 3B), the trunk and the ankle (R = −0.633, *p* < 0.001, Figure 3C), and the knee and the ankle (R = 0.664, *p* < 0.001, Figure 3F). The hip was not significantly correlated with any of the other joints (Figures 3A, D, and E).

## Discussion

This study focused on the kinematics in both the AP and vertical directions during squatting, which is a postural strategy or may be used as a training exercise in the standing posture. Squat performance has been assessed via the knee angle and marker positions in previous studies (Bagwell et al., 2016; Kim et al., 2015; Schoenfeld, 2010; Zawadka et al., 2020). Squats can be divided into three subtypes based on the knee flexion angle: 1) semi squatting (40°), 2) half squatting (70° to 100°), and 3) deep squatting (>100°) (Dionisio et al., 2008; Escamilla et al., 2001; Schoenfeld, 2010). Moreover, Kumar et al. (2014) defined a deep squat as a descent and an ascent of 25% of the participant’s height. Although Escamilla et al. (2001) reported that the maximal knee flexion and maximal squat depth were approximately (but not over) 100° and approximately 26.5% of the participant’s height, respectively, during deep squatting among masters’ level powerlifters, their squats did not seem to satisfy the two conditions of deep squats described above. The squat depths for the participant’s height and the maximal angle of knee flexion in the current study were approximately 22.8% and 100°, respectively, both of which satisfied the criteria for half squats.

Previous studies (Kim et al., 2015; Zawadka et al., 2020) of the relationship between ROMs of the trunk or lower limb joints and squat depth showed that all joints were related to squat depth. However, these studies did not clearly demonstrate how joint motion controlled the COM during squats. From the results of each joint ratio for the squat depth with the COM, the knee expanded by a greater angle to achieve the squat depth than the trunk, hip, and ankle joints. Although this result was obtained from the maximum angle of each ROM, it indicates that the knee has a high predictive value for squat depth and may play a fine-tuning role in the COM descent from a motor control viewpoint. Therefore, our study confirms that the knee is the key joint (i.e., the joint that defines motion) in functional squatting for vertical postural strategy, as documented in previous studies (Kasahara et al., 2015; Kim et al., 2017; Nashner et al., 1985).

Despite the absence of a significant correlation between squat depth and ankle ROM, ankle ROM was revealed as a significant factor in the multivariate linear regression analysis. In this case, ankle ROM was considered a suppressor variable (Friedman et al., 2005). Although this relationship should be interpreted with caution, these results may be related to the role of AP postural control accompanying the squat descent (i.e., the ankle strategy) (Kasahara et al., 2015; Nashner et al., 1985). We speculate that two mechanisms underlie the COM descent: lower limb control centered on the knee, and trunk control that is the weight of more than 50% combining the head, the neck, and both arms (de Leva et al., 1996). Previous studies have shown that the COP shifts forward during squats (Dionisio et al., 2008; Kuo et al., 2011; Schoenfeld, 2010). In general, the AP shifts in the COP and COM during the standing posture are controlled by the hip and ankle joints (Kasahara et al., 2015; Nashner et al., 1985). The correlation between AP displacement of the COM and the ankle and hip joints supports the postural strategy during standing (Dionisio et al., 2008; Kasahara et al., 2015; Nashner et al., 1985) and suggests that ankle dorsiflexion and hip flexion shift the COM in the anterior and posterior directions, respectively. Fuglsang et al. (2017) also documented that ankle dorsiflexion had a negative relationship with trunk flexion, indicating a tradeoff. Previous studies that defined squat depth according to the joint angle and the body marker position could not propose the potential roles of each joint during squatting (Bagwell et al., 2016; Kim et al., 2015; Zawadka et al., 2020). Therefore, the COM parameters in this and a previous study (Stevens et al., 2018) are valuable for the assessment of squats.

The ankle was the first to reach its maximal angle in this study, staying at that angle while the other joints (i.e., the knee and hip) continued their motion for a very short period of time. The sequence of joint motion in this study was consistent with that in a previous study (Zawadka et al., 2020). Originally, the ROM of ankle dorsiflexion was smaller than that of the other joints because dorsiflexion of the ankle joint (i.e., the talocrural joint) was limited by the collision between the talus and tibia. Furthermore, the ankle motion reaches its approximate end point because the trochlea of the talus is tucked between the lateral malleolus of the fibula and the medial malleolus of the tibia. In this study, we selected half squats based on feedback from coaches and physical therapists in clinical settings (Ishida et al., 2022a, 2022b, 2023). In the deep squat following the half squat, the contribution of other joint motions except for the ankle would be necessarily increased more because the ankle motion is limited by the anatomical motion restrictions of the ankle. Furthermore, with increasing squat depth (i.e., deep squatting), the horizontal posterior displacement and posterior tilt of the pelvis increased, and the COM shifted backward (Swinton et al., 2012). In cases where heel-off is not permitted, the ankle motion turns from dorsiflexion to plantarflexion (Dionisio et al., 2008; Schoenfeld, 2010). The ankle may reach its maximal angle and turn its direction of motion earlier in individuals with ankle ROM restriction. Therefore, during squatting, the ankle is considered another key joint along with the knee.

This study has several limitations. First, participants included in this study were limited to young adults, and future studies should examine other age groups. Second, the squats in this study were near half-squats or parallel squats with both legs and differed from other squats (e.g., quarter squat, partial squat, full squat, and deep squat). Additional studies assessing the COM, muscle activities, and joint moments should be conducted to investigate the role of each joint motion in other squatting movements.

## Conclusions

The current study revealed the relationship between the COM and related joints during the descent phase of squats. Ankle dorsiflexion reached its maximum angle first among the examined joints. The knee plays a key role in the COM descent. In addition, the ankle and the hip seem to contribute to controlling the COM in the AP direction. Each lower limb joint plays a different functional role in postural control during squats.
